# Overexpressed ITGA2 promotes malignant tumor aggression by up-regulating PD-L1 expression through the activation of the STAT3 signaling pathway

**DOI:** 10.1186/s13046-019-1496-1

**Published:** 2019-12-09

**Authors:** Dianyun Ren, Jingyuan Zhao, Yan Sun, Dan Li, Zibo Meng, Bo Wang, Ping Fan, Zhiqiang Liu, Xin Jin, Heshui Wu

**Affiliations:** 1 Department of Pancreatic Surgery, Union Hospital, Tongji Medical College, Huazhong University of Science and Technology, Wuhan, 430022 China; 2 Sino-German Laboratory of Personalized Medicine for Pancreatic Cancer, Union Hospital, Tongji Medical College, Huazhong University of Science and Technology, Wuhan, 430022 China; 3 Cancer Center, Union Hospital, Tongji Medical College, Huazhong University of Science and Technology, Wuhan, 430022 China; 4 Cardiovascular medicine department, Union Hospital, Tongji Medical College, Huazhong University of Science and Technology, Wuhan, 430022 China

**Keywords:** Integrin α2, Programmed death-ligand 1, Phosphorylation of STAT3, Cancer

## Abstract

**Background:**

Recent studies have reported that Integrin alpha 2 (ITGA2) plays an essential role in tumor cell proliferation, invasion, metastasis, and angiogenesis. An abnormally expressed ITGA2 correlates with unfavorable prognoses in multiple types of cancer. However, the specific mechanism of how ITGA2 contributes to tumorigenesis remains unclear.

**Methods:**

The GEPIA web tool was used to find the clinical relevance of ITGA2 in cancer, and this significance was verified using Western blotting analysis of paired patient tissues and immunohistochemistry of the pancreatic cancer tissue. Functional assays, such as the MTS assay, colony formation assay, and transwell assay, were used to determine the biological role of ITGA2 in human cancer. The relationship between ITGA2 and programmed death-ligand 1 (PD-L1) was examined using Western blot analysis, RT-qPCR assay, and immunohistochemistry. The protein-protein interaction between ITGA2 and STAT3 was detected via co-immunoprecipitation.

**Results:**

Our study showed that ITGA2 was markedly overexpressed in several malignant tumor cells and clinical tissues. Blocking ITGA2 inhibited the proliferation and invasion ability of cancer cells significantly, whereas overexpressed ITGA2 increased the degree of those processes considerably. Additionally, the RNA-seq assay indicated that ITGA2 transcriptionally regulated the expression of PD-L1 in pancreatic cancer. We also demonstrated that ITGA2 interacted with STAT3 and up-regulated the phosphorylation of STAT3; this interaction might involve the mechanism of ITGA2 inducing PD-L1 expression in cancer cells. Our results suggest that ITGA2 plays a critical role in cancer cell progression and the regulation of PD-L1 by activating the STAT3 pathway.

**Conclusions:**

We identified a novel mechanism by which ITGA2 plays a critical role in modulating cancer immune response by transcriptionally increasing the expression of PD-L1 in cancer cells. Thus, targeting ITGA2 is an effective method to enhance the efficacy of checkpoint immunotherapy against cancer.

## Introduction

According to findings in the Global Health Observatory data repository from 2011, malignant tumors caused more deaths than coronary heart diseases or stroke (http://apps.who.int/gho/data/node.main.CODWORLD?lang=en). There were 14.1 million new cases and 8.2 million deaths from cancers in 2012 [1]. Thus, studying the pathogenesis of malignant tumors is essential for identifying more therapeutic targets for cancer treatment.

Integrin alpha 2 (ITGA2) is the alpha subunit of a transmembrane receptor for collagens and related proteins [2]. ITGA2 frequently forms a heterodimer α2β1 with a β subunit, which mediates the adhesion of platelets and other cells to the extracellular matrix (ECM) [3, 4]. ITGA2 is overexpressed in several types of tumors, such as pancreatic cancer, gastric cancer, liver cancer, prostate cancer, and breast cancer [5–9]. Recently, increasing evidence has suggested that ITGA2 might play an essential role in modulating tumor cell migration, invasion, and metastasis [10, 11]. However, the specific mechanism of how ITGA2 is involved in all this is still confusing. Reportedly, the loss of ADAR1 could lead to the up-regulation of ITGA2 in hepatocellular carcinoma [12]. Besides, the loss of ADAR1 overcomes resistance to immune checkpoint blockade in tumors [13]. Therefore, we hypothesized that ITGA2 might also regulate immune checkpoint blockade responses in tumors.

The relationship between host and tumor is dynamic, and tumors can often escape from immune recognition and immune attack, which determines the clinical course of cancers. The aberrant activation of immune checkpoints is one of the characteristics of cancer cells escaping from host immune salience [14, 15]. Importantly, the programmed cell death protein 1/programmed cell death-ligand 1 (PD-1/PD-L1) pathway is one of the most studied immune checkpoint pathways to inactivate immune responses in the tumor microenvironment [16]. Several studies have demonstrated that blocking PD-L1 can improve the immune functions of T cells in many malignant tumors [17–20]. The PD-1/PD-L1 interaction could inhibit T cell response by inducing the apoptosis of CD8^+^ T cells and promoting CD4^+^ T differentiating to regulatory T cells [21]. Moreover, PD-L1 expressed on the surface of malignant tumor cells could directly suppress the antitumor activity of CD8^+^ T cells [22]. Thus, the way to regulate PD-L1 expression levels in cancer cells is crucial to exploring novel therapeutic strategies to improve the immune checkpoint blockade effect on cancers.

In our current study, we showed that the expression level of ITGA2 was highly related to the ability of proliferation and invasion of tumor cells. We further revealed that ITGA2 interacted with STAT3 and up-regulated PD-L1 expression by increasing the phosphorylation of STAT3 in cancer cells. Collectively, these data suggest that ITGA2 could be a potential target for improving the anti-tumor efficiency of immune checkpoint-based therapies in cancer.

## Materials and methods

### Cell culture

The PANC-1, HepG2, SGC-7901, and MDA-MB-231 cell lines were purchased from ATCC. The Panc02 cell line was acquired from Tong Pai Technology (Shanghai, China). PANC-1, HepG2, and MDA-MB-231 cell lines were cultured in DMEM (Thermo Fisher Scientific, USA) supplemented with 10% FBS (HyClone, USA) at 37 °C in a 5% CO_2_ incubator. SGC-7901 cells were cultured in RPMI 1640 containing 10% FBS under similar conditions.

#### Antibodies and plasmids

Human expression vectors for flag-ITGA2 recombinant proteins were generated using the pcDNA3.1 backbone vector. The ITGA2 antibody (ab133557, 1:1000) was purchased from Abcam; GAPDH (10494–1-AP, 1:3000) from Proteintech; STAT3 (10253–2-AP, 1:1000) from Proteintech; PD-L1 (13684S, 1:1000) from Cell Signaling Technology; and Phospho-STAT3-Y705 (abs118973, 1:1000) from Absin.

### RNA interference

The sh-Control and sh-ITGA2s were procured from Sigma-Aldrich. Lipofectamine 3000 (Invitrogen, USA) and Opti-MEM media (Invitrogen, USA) were used for the transfection reactions; lipofectamine 3000 was used to transfect 293 T cells to shRNA plasmids and viral packaging plasmids (pVSV-G and pEXQV). 24 h after transfection, the medium was replaced with fresh DMEM containing 10% FBS and 1 mM sodium pyruvate, and 48 h post-transfection, the virus culture medium was collected and added to the PANC-1, HepG2, SGC-7901, and MDA-MB-231 cells supplemented with 12 μg/ml of polybrene. 24 h after infection, the infected cells were selected with 10 μg/ml of puromycin. The shRNA sequence information is provided in the Additional file 1: Table S2.

### Western blot analysis

Written informed consent was obtained from patients before surgery, as described previously [23]. The use of human tissue was approved by the Ethics Committee of Tongji Medical College, HUST, China.

Cell lysates were prepared using a radioimmunoprecipitation assay (RIPA) buffer (Thermo Fisher Scientific, USA) in the presence of a protease inhibitor cocktail (Sigma-Aldrich, USA) and Halt phosphatase inhibitor cocktail (Thermo Scientific, USA). Protein concentration was determined using a protein quantification kit (Sigma-Aldrich, USA) to ensure equal amounts of total protein were loaded in each well of SDS-PAGE gels. The protein was transferred to PVDF membranes (Pierce Biotechnology, USA) eventually and blocked with 5% not-fat milk for 1 h at room temperature (RT), then incubated with the specific primary antibody at 4 °C overnight or at room temperature (RT) for 2 h and washed with PBS for 3 times, followed by another incubation with secondary antibodies (BOSTER, USA). Finally, after washing with PBS for 3 times, the membranes were exposed to X-ray films using ECL detection reagents (Thermo Fisher Scientific, USA).

### Quantitative RT-PCR assay

Total RNA was prepared using a Trizol reagent (Invitrogen, 15,596,026, USA). RNA samples (1 μg) were reverse-transcribed using a PrimeScript™ RT reagent Kit (TAKARA, RR047A, JPN). Quantitative real-time PCR was performed using a TB Green™ Fast qPCR Mix kit (TAKARA, RR430A, JPN). The sequences of the primers used for qRT-PCR are shown in the Additional file 1: Table S1. Values represent the averages of three technical replicates from at least three independent experiments (biological replicates).

### Immunohistochemistry (IHC)

Tissue microarray slides were purchased from Outdo Biobank (Shanghai, China) (HPan-Ade060CD-01). IHC analysis was performed with ITGA2 (Abcam, 1:5000), PD-L1 (CST, 1: 1000), and Phospho-STAT3-Y705 (Absin, 1:2000) antibodies to determine their protein expression levels. Two independent pathologists, who were uninformed about the patient data and histopathological features of the samples, were responsible for reviewing and scoring the degree of immunostaining separately. Staining intensity scoring was done, as described previously [24].

### Colony formation assay

The colony formation assay was performed to examine the biological effect of ITGA2 on tumor cell survival. 500 Tumor cells infected with sh-Control or sh-ITGA2s, pcDNA3.1, or flag-ITGA2 plasmids were plated in six-well plates, and colony formation assays were performed and photographed after 7–14 days of plating.

### Cell invasion and migration assay

The transwell assay was employed to evaluate cell invasion using 24-well Corning Costar inserts with 8-μm pores precoated with Matrigel (BD, United States; diluted 1: 8) for 6 h in an incubator. To begin with, 1 × 10^4^ cancer cells were planted into upper invasion chambers without FBS, and DMEM containing 30% FBS was introduced to the lower chambers. After a 12 to 24-h culture, cells were fixed on the insert membranes using methanol and stained with a Crystal Violet Staining Solution (Solarbio, China). The invading cells were photographed and evaluated under a microscope at five fields per well.

Transwell assay was employed again to examine cell migration using 24-well Corning Costar inserts with 8-μm pores without Matrigel-coating. Initially, 1 × 10^4^ cancer cells were planted into upper chambers, and DMEM containing 30% FBS was placed in the lower chambers. After a 12 to 24-h culture, the cells were fixed on the insert membranes using methanol and stained with a Crystal Violet Staining Solution (Solarbio, China). The migrated cells were photographed and assessed under a microscope at five fields per well.

### MTS assay

The proliferation ability of tumor cells was assessed using (3-(4,5-dimethylthiazol-2-yl)-5-(3-carboxymethoxyphenyl)-2- (4-sulfophenyl)-2H-tetrazolium) (MTS reagent) (Abcam, ab197010, USA). Briefly, 1000 cells were introduced into 96-well plates with 100 μl DMEM containing 10% FBS and treated with serial small molecular inhibitors under different concentration gradients. 20 μl of an MTS reagent was added to each well three hours before the end of the incubation period following the manufacturer’s instructions. The absorbance of each well was detected at 490 nm with a microplate reader.

### Flow cytometry

PANC-1, HepG2, SGC-7901, and MDA-MB-231 cells infected with sh-Control or sh-ITGA2s were prepared and stained for flow cytometry with the following antibodies: isotype APC anti-human IgG Fc Antibody (Biolegend, clone HP6017, USA) or APC anti-human CD274 antibody (Biolegend, clone 29E.2A3, USA) or for 30 mins at 4 °C. The cells were then washed with PBS three times, resuspended in 150 μl staining buffer, and analyzed for flow cytometry.

For flow cytometry analysis of the mouse tissue samples, single-cell suspensions were prepared and stained for flow cytometry with the following antibodies: APC conjugated CD45 antibody (Biolegend, 103,112, USA); FITC conjugated CD4 antibody (Biolegend, 100,510, USA); PE-conjugated CD8 antibody (Biolegend, 100,708, USA); APC conjugated CD11b antibody (Biolegend,101,212, USA); and FITC conjugated Gr1 antibody (Biolegend, 108,406, USA). Flow cytometry was performed on BD FACSCelesta (BD Biosciences, USA), and the data were analyzed using FlowJo.

### Bioinformatics mining

GEPIA (http://gepia.cancer-pku.cn/) and Human Protein Atlas cancer databases (https://www.proteinatlas.org/) were mined to predict the ITGA2 differential expression level in cancerous and healthy groups. The survival analyses, performed by GEPIA for hypothesis evaluation, used a log-rank test based on gene expression levels. Gene correlation analyses conducted by Pearson correlation statistics were carried out with GEPIA for all the given sets of GTEx and TCGA expression data.

### Generation of PDAC xenografts in nude mice

Athymic nude (nu/nu) mice (4–5 weeks old, male) were purchased from Vitalriver (Beijing, China). 5 × 10^6^ PANC-1 human pancreatic cancer cells infected with sh-Control or sh-ITGA2 lentivirus were dispersed in a 100 μl solution of PBS and then inoculated subcutaneously into the left dorsal of the nude mice. Tumor sizes were measured with a digital Vernier caliper every two days for a total of 21 days. Tumor volumes were calculated using the formula: tumor volume (mm^3^) = (L x W^2^)/2. The animals were sacrificed on day 21 or when tumor volume reached 1000 mm^3^. The experimental procedures with nude mice were conducted per the guidelines approved by our local ethics committee (Tongji Medical College, HUST, China).

### Syngeneic tumor model treatment protocol

All experimental procedures with nude mice were conducted per the guidelines approved by the local ethics committee (Tongji Medical College, HUST, China). C57BL/6 mice (6–8 weeks male, Vitalriver, Beijing, China) were subcutaneously injected at the right flank with 5 × 10^6^ Panc02 cells infected with sh-Control or sh-ITGA2 #1 lentivirus and diluted in 100 μl solution (PBS and Matrixgel, 1:1 ratio). Tumor lengths and widths were measured every day with a digital caliper, and tumor volumes were calculated using the formula: (L x W^2^)/2. When the tumor size reached approximately 50 mm^3^, mice with analogous types of tumors were divided randomly into two groups and injected intraperitoneally with 200 μg of anti-PD-1 antibody (BioXcell, Clone RMP1–14, USA) or 200 μg of IgG (BioXcell, Clone 2A3, USA) (given at days 0, 3, 6). Mice were euthanized once the tumor volumes reached 200 mm^3^, and their tumors were harvested.

### Immunoprecipitation

A 0.5–2.0 μg primary antibody was added to the cell lysate sample and incubated on ice overnight. The next day, 20–50 μL of Protein A beads (CST, #9863, USA) was added to the cell lysate and primary antibody mixture, and the content was gently rotating incubated at 4 °C for 3 h. The next day, the beads were washed at least six times with lysate buffer on ice, and then subjected to western blotting analysis.

### Statistical analysis

GraphPad Prism 6 software (GradPad Software, Inc) was used for all statistical analyses. Statistical significance was assessed using the paired t-test, Student’s t-test, and one or two-way ANOVA, followed by Tukey’s multiple comparison tests. Only *P* values less than 0.05 were considered significant. All the values are expressed as the mean ± SD.

## Result

### The overexpression of ITGA2 correlates with unfavorable prognoses in malignant tumors

**Fig. 1:**
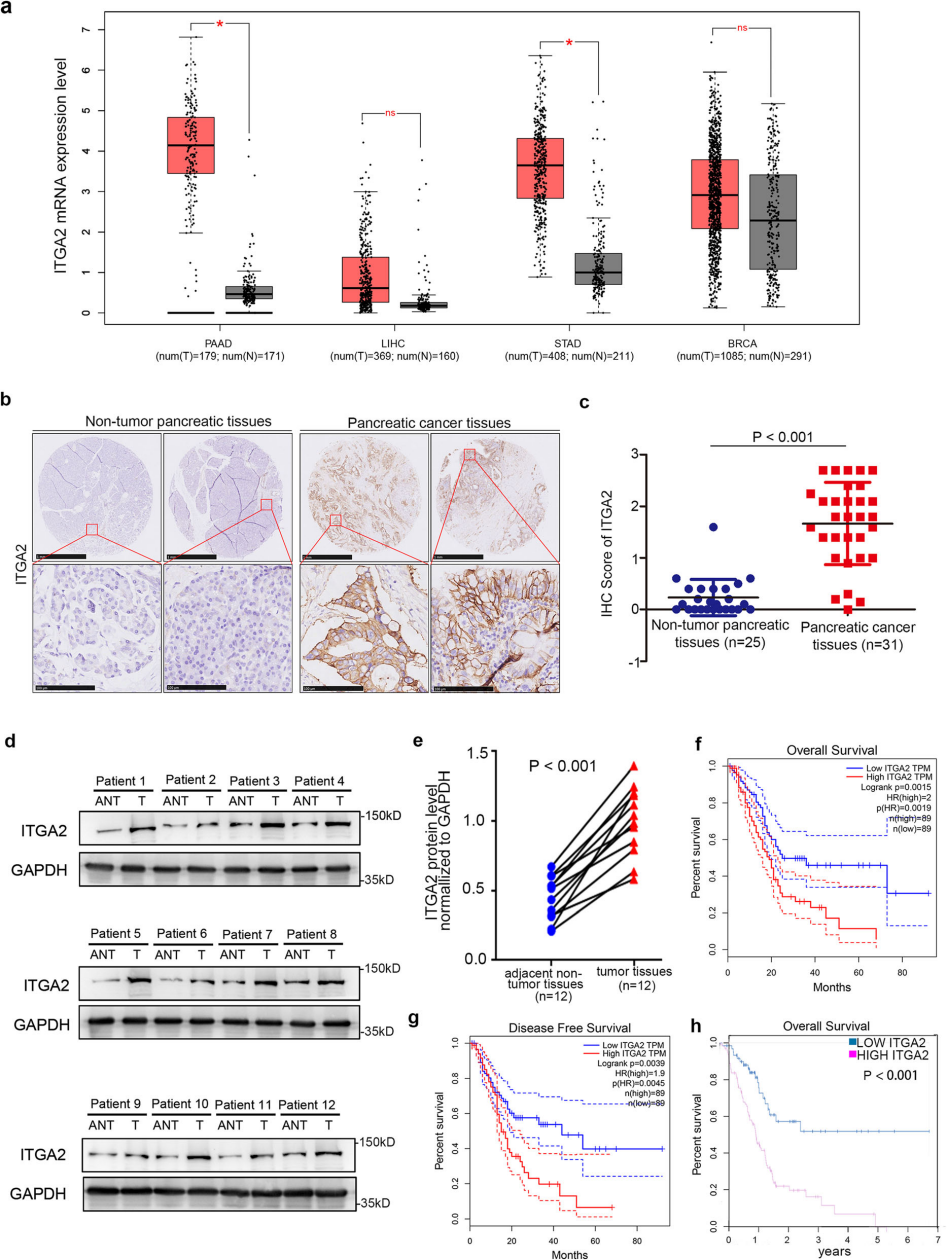
The overexpression of ITGA2 is correlated with unfavorable prognosis in malignant tumors. **a**. ITGA2 mRNA expression level determined by the GEPIA web tool. The boxplot analysis showed the expression level by log2 (TPM + 1) on a log-scale. ns, not significant; *, *P* < 0.05. **b**. IHC Images of ITGA2 staining using TMA tissue sections. The scale bars were shown in the figure. **c**. Dot plots to show the IHC score of ITGA2 expression using TMA tissue sections (normal pancreatic specimens: *n* = 25, PDAC TMA specimens: *n* = 31, *P* < 0.001). Statistical analyses were performed with D’Agostino & Pearson omnibus normality test. **d** and **e**. Western blot analysis for the expression of ITGA2 in 12 paired primary PDAC tissues (T) and the matched adjacent normal tissues (ANT) of the same patient (d). The proteins expression level of ITGA2 were quantified by ImageJ software (e). GAPDH served as an internal reference. The *P* values were also shown. Statistical analyses were performed with D’Agostino & Pearson omnibus normality test. **f** and **g**. GEPIA web tool was searched for the overall survival (f, *P* = 0.015, HR = 2.0) and disease-free survival (**g**, *P* = 0.039, HR = 1.9) of PDAC patients. **h**. The Human Protein Atlas database was searched for the overall survival of PDAC patients (*P* < 0.001)

After analyzing the mRNA expression level of ITGA2 in several cancers and non-tumor tissues using the GEPIA web tool, we found that the mRNA expression level of ITGA2 was significantly up-regulated in pancreatic adenocarcinoma (PAAD) and stomach adenocarcinoma (STAD) (Fig. 1)a.

Since the differential expression of ITGA2 was more significant in pancreatic cancer compared with non-tumor tissue, we paid more attention to exploring the biological role of ITGA2 in PDAC. The protein expression levels of ITGA2 in PDAC specimens were examined. Immunohistochemistry (IHC) based on a cohort of patients (healthy pancreatic specimens: *n* = 25, PDAC TMA specimens: *n* = 31) was performed using the tissue microarray approach (TMA). The IHC staining score was evaluated, as described previously [23], and the results showed that ITGA2 proteins increased profoundly in pancreatic cancer tissues compared with non-tumor pancreatic tissues (Fig. 1)b,c. Consistent with IHC, comparative analysis with matched non-tumor pancreatic tissues demonstrated that ITGA2 was overexpressed in twelve primary PDAC samples (Fig. 1)d,e.

To confirm the clinical relevance of ITGA2 expression levels on survival rate in malignant tumors further, the overall survival (OS) and disease-free survival (DFS) of several cancer patients related to ITGA2 expression were examined using the GEPIA web tool and The Human Protein Atlas web tool. The results revealed that the high expression of ITGA2 was significantly associated with unfavorable survival rates in PAAD and liver hepatocellular carcinoma (LIHC) patients, although the outcome was more evident in PDAC patients (Fig. 1f-h and Additional file 1: S1a-c). These findings suggest that ITGA2 is up-regulated in PAAD and STAD and is related to poor prognoses in PAAD and LIHC patients.

### Silencing ITGA2 suppresses cancer aggression ability in vitro

**Fig. 2:**
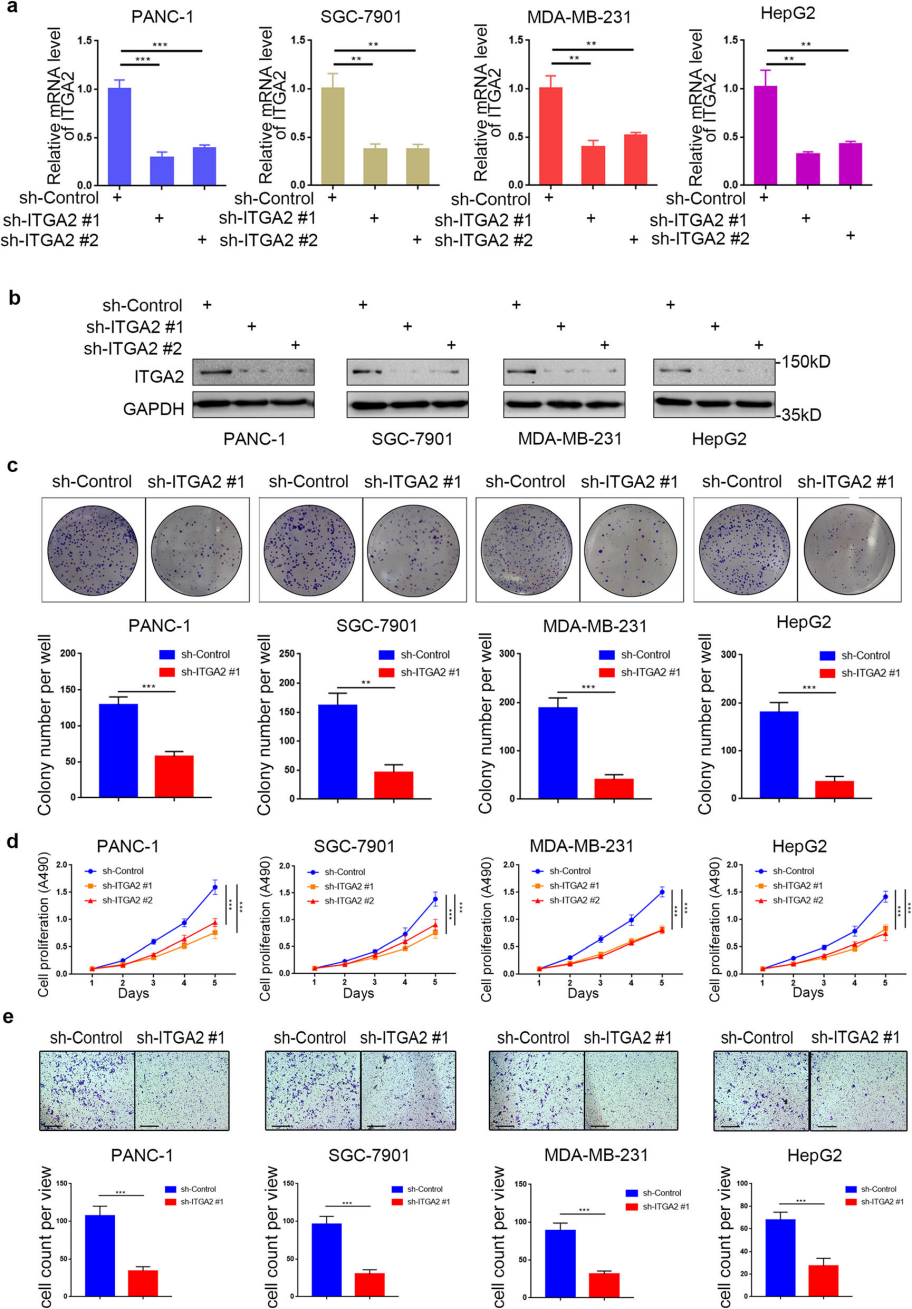
Silencing ITGA2 suppresses the aggressive ability of malignant cancer in vitro **a** and **b**. RT-PCR (**a**) and Western blot analysis (**b**) of ITGA2 expression in PANC-1, HepG2, SGC-7901, and MDA-MB-231 cells infected with sh-Control or sh-ITGA2s. GAPDH served as an internal reference. Data presented as the mean ± SD of three independent experiments. Each sh-ITGA2 group was compared with sh-Control group. Statistical analyses were performed with one-way ANOVA followed by Tukey’s multiple comparison’s tests. **, *P* < 0.01; ***, *P* < 0.001. **c**-**e**. PANC-1, HepG2, SGC-7901, and MDA-MB-231 cells were infected with sh-Control or sh-ITGA2 #1. The cells were harvested for colony formation assay (**c**), MTS assay (**d**), and migration assay (**e**) after forty-eight hours culturing. For e, representative images of migrated cells were shown based on a transwell assay. Each bar represents the mean ± SD of three independent experiments. Each sh-ITGA2 group was compared with sh-Control group. Statistical analyses were performed with one-way ANOVA followed by Tukey’s multiple comparison’s tests.. **, *P* < 0.01; ***, *P* < 0.001

Since up-regulated ITGA2 was a poor prognostic biomarker for cancer, we explored its biological role in cancerous tumors further. Following the knockdown of ITGA2 using short Harpine RNA (shRNA) in PANC-1, HepG2, SGC-7901, and MDA-MB-231 cell lines (Fig. 2)a,b, the MTS and colony formation assays demonstrated that silencing ITGA2 inhibited the cancer cell growth (Fig. 2)c,d. Additionally, the transwell assay showed that the migration and invasion abilities of cancer cells decreased markedly after ITGA2 down-regulation (Fig. 2e and Additional file 1: S2). Our results indicate that knocking down ITGA2 could inhibit cancer aggression in vitro.

### Overpressed ITGA2 promotes cancer aggression ability in vitro

**Fig. 3:**
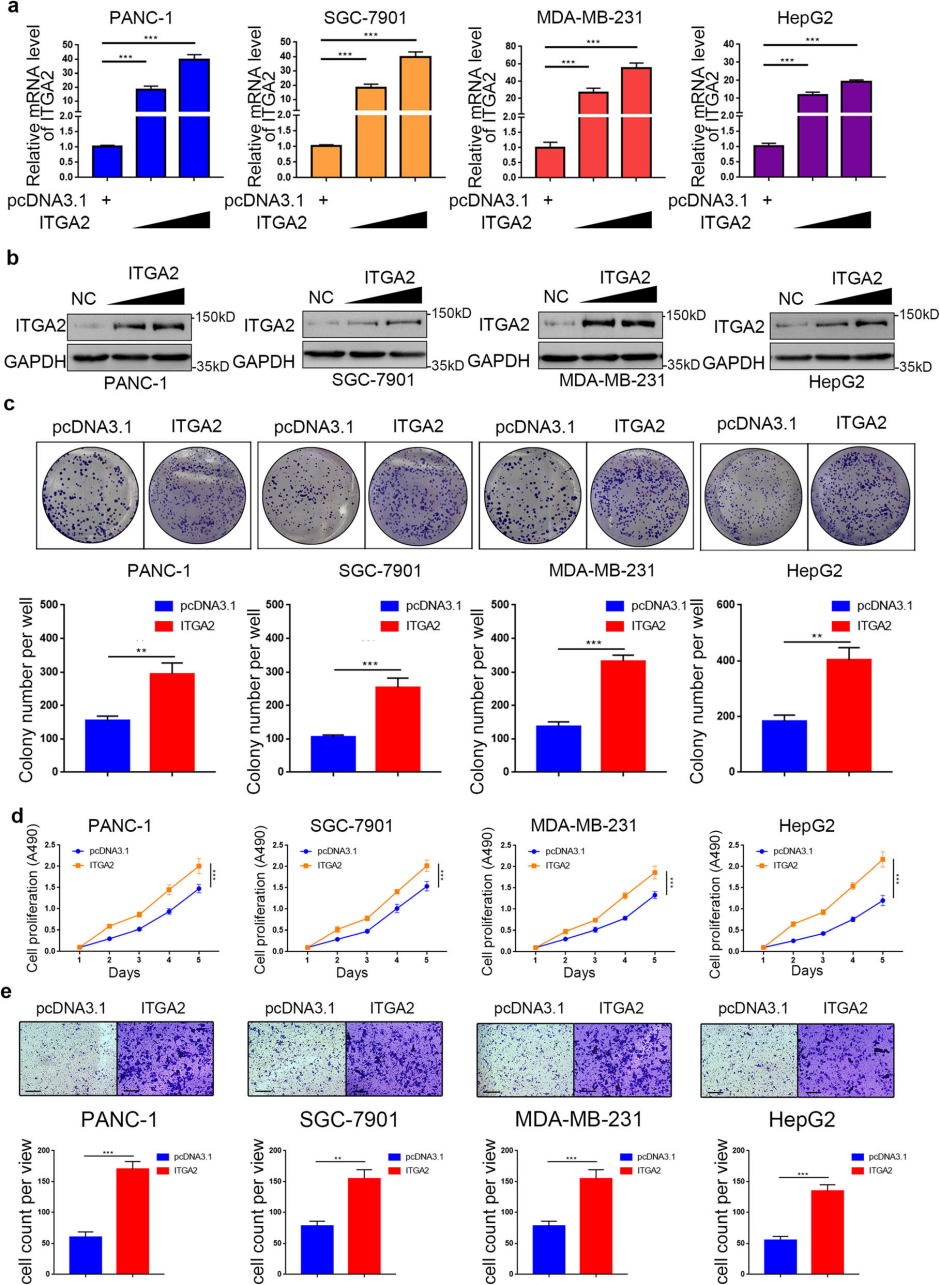
Overpressed ITGA2 promotes the aggressive ability of malignant cancer in vitro. **a** and **b**. RT-PCR (**a**) and Western blot analysis (**b**) of ITGA2 expression in PANC-1, HepG2, SGC-7901, and MDA-MB-231 cells with normal or stably overexpressed ITGA2. GAPDH served as an internal reference. Data presented as the mean ± SD of three independent experiments. ITGA2 overexppression groups were compared with pcDNA 3.1 transfection group. Statistical analyses were performed with one-way ANOVA followed by Tukey’s multiple comparison’s tests. Compared groups were shown in the figures. ***, *P* < 0.001. **c**-**e**. PANC-1, HepG2, SGC-7901, and MDA-MB-231 cells were infected with pcDNA3.1 or ITGA2 plasmid. The cells were harvested for colony formation assay (**c**), MTS assay (**d**), and migration assay (**e**) after forty-eight hours culturing. For e, Representative images of migrated cells were shown based on a transwell assay. Each bar represents the mean ± SD of three independent experiments. ITGA2 overexppression groups were compared with pcDNA 3.1 transfection group. Statistical analyses were performed with one-way ANOVA followed by Tukey’s multiple comparison’s tests. Compared groups were shown in the figures. **, *P* < 0.01; ***, *P* < 0.001

To explore the role of overexpressed ITGA2 in cancer progression further, PANC-1, HepG2, SGC-7901, and MDA-MB-231 cell lines with stably overexpressed ITGA2 were established (Fig. 3)a,b. Colony formation and MTS assays indicated that overexpressed ITGA2 enhanced the growth capability of PANC-1 cells, HepG2 cells, SGC-7901 cells, and MDA-MB-231 cells (Fig. 3)c,d. Moreover, the transwell assay showed that migration and invasion abilities were also up-regulated in ITGA2-overexpressing cancer cells (Fig. 3e and Additional file 1: S3). These data suggest that the overexpression of ITGA2 could promote the aggression ability of cancer cells in vitro.

### ITGA2 promotes tumor growth in pancreatic cancer in vivo

**Fig. 4:**
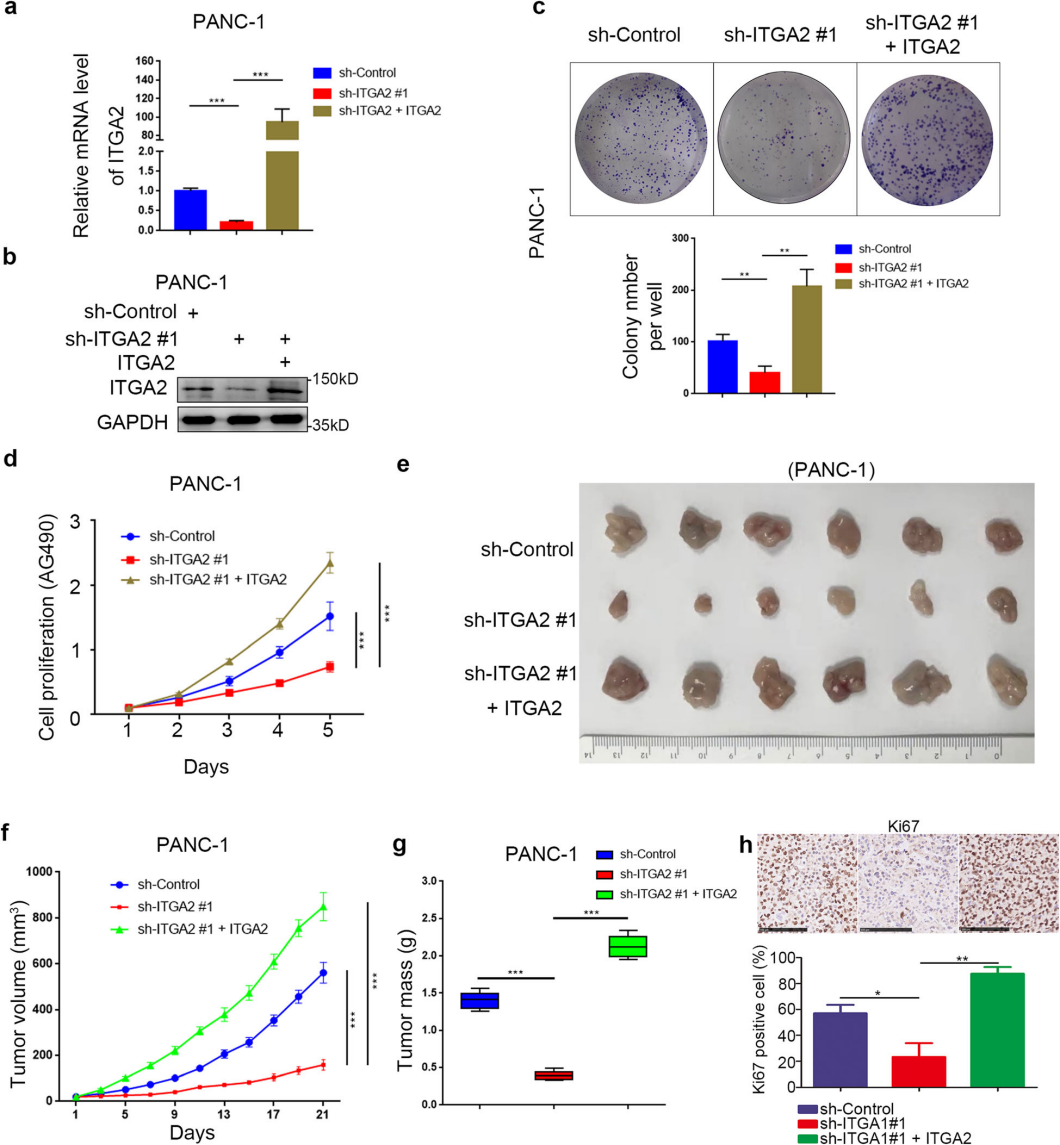
ITGA2 promoted tumor growth in pancreatic cancer in vivo. **a** and **b**. PANC-1 cells were infected with sh-Control, sh-ITGA2, or sh-ITGA2 and ITGA2 plasmid. The cells were harvested for RT-PCR analysis (**a**) and Western blot analysis (**b**). GAPDH served as an internal reference. sh-Control group was compared with sh-ITGA2 group, sh-ITGA2 group was compared with sh-ITGA2 + ITGA2 group. Statistical analyses were performed with one-way ANOVA followed by Tukey’s multiple comparison’s tests. ***, *P* < 0.001. **c** and **d**. PANC-1 cells infected with sh-Control, sh-ITGA2, or sh-ITGA2 and ITGA2 plasmid were harvested for colony formation assay (**c**) and MTS assay (**d**) after forty-eight hours culturing. Each bar represents the mean ± SD of three independent experiments. sh-Control group was compared with sh-ITGA2 group, sh-ITGA2 group was compared with sh-ITGA2 + ITGA2 group. Statistical analyses were performed with one-way ANOVA followed by Sidak’s multiple comparison’s tests. **, *P* < 0.01. **e**-**g**. After 72 h postinfection, PANC-1 cells infected with sh-Control, sh-ITGA2, or sh-ITGA2 and ITGA2 were subcutaneously injected into nude mice. The tumors were harvested and photographed (**e**) on day 21. Data on tumor volume (**f**) and tumor mass (**g**) were shown as means ± SD (*n* = 5). sh-Control group was compared with sh-ITGA2 group, sh-ITGA2 group was compared with sh-ITGA2 + ITGA2 group. Statistical analyses were performed with two-way ANOVA followed by Sidak’s multiple comparison’s tests. ns, not significant; ***, *P* < 0.001. **h**. IHC analysis for Ki-67 expression was performed in tumors harvested from xenografts, and percents of the Ki-67 positive cells were quantified. All data were shown as mean ± SD (n = 5). sh-Control group was compared with sh-ITGA2 group, sh-ITGA2 group was compared with sh-ITGA2 + ITGA2 group. Statistical analyses were performed with one-way ANOVA followed by Tukey’s multiple comparison’s tests. *, *P* < 0.05; **, *P* < 0.01

Given that ITGA2 levels regulated the progression of cancer cells in vitro, we studied the tumor-growth-promoting effect of ITGA2 in pancreatic cancer in vivo. PANC-1 cells infected with sh-Control, sh-ITGA2, or sh-ITGA2 + ITGA2 were harvested for RT-PCR analysis, Western blot analysis, colony formation assay, and MTS assay (Fig. 4)a,d. The colony formation and MTS assays revealed that the inhibition of ITGA2 significantly slowed down tumor growth, whereas ITGA2 overexpression promoted cancer growth in PANC-1 cells (Fig. 4)c,d.

PANC-1 cells under the same conditions as above were subcutaneously injected into the left flank of nude mice for the xenograft assay. As Fig. 4e-g shows, tumors formed in ITGA2-silenced PANC-1 cells were smaller and lighter than those in ITGA2 expressed cells, but the body weights were not significantly different between the different treatments (Additional file 1: Fig. S4a). In turn, tumors formed after rescuing ITGA2 were more massive than tumors from ITGA2-knocked down cells. Moreover, Ki-67 staining of the implanted tumors further showed that cell proliferation ability correlated significantly with the expression level of ITGA2 (Fig. 4h). Therefore, ITGA2 could promote tumor growth in pancreatic cancer in vivo.

### ITGA2 transcriptionally increases PD-L1 expression in solid cancer cells

**Fig. 5:**
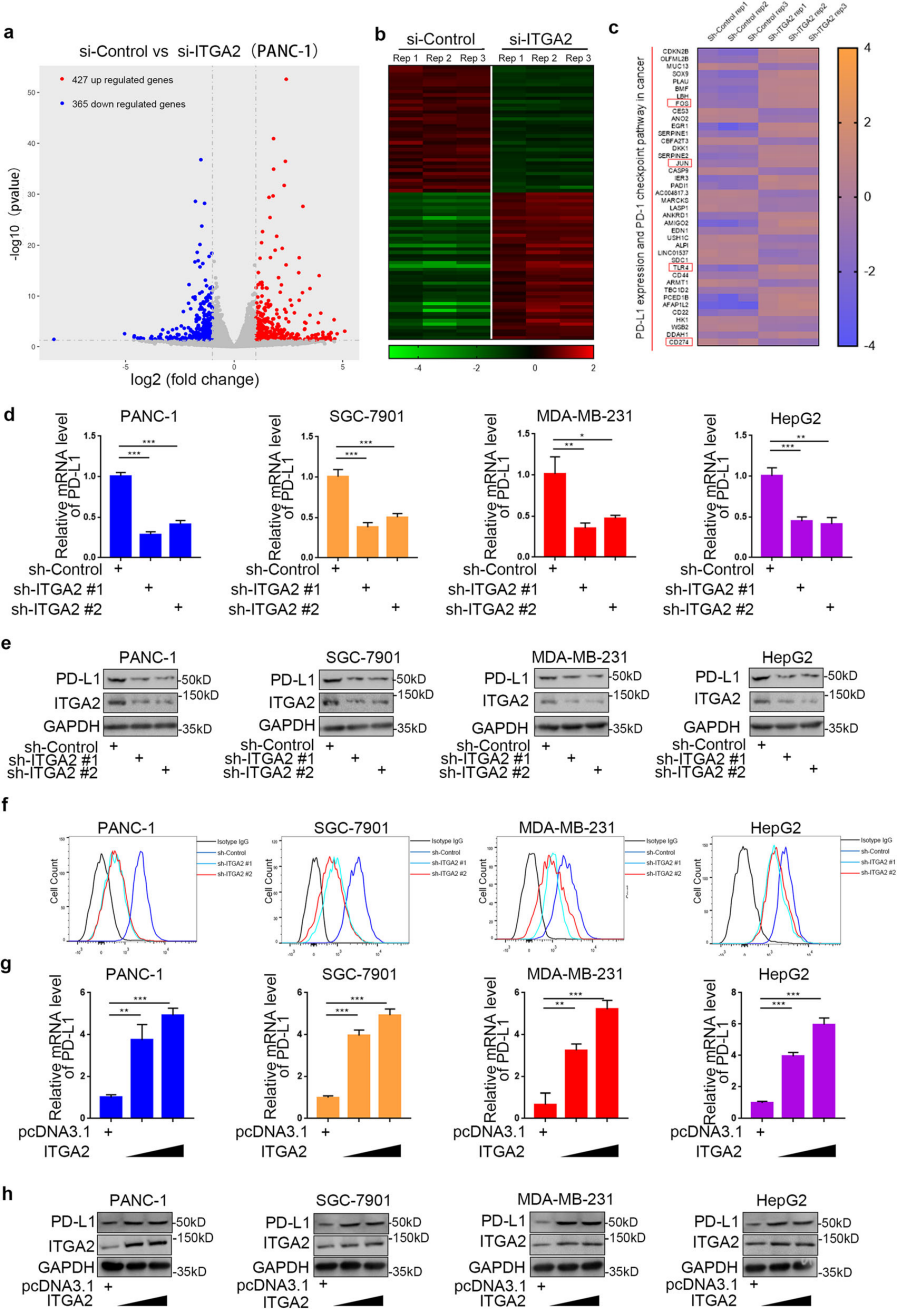
ITGA2 transcriptionally increases PD-L1 expression in solid cancer cells. **a** and **b**. Volcano plot (**a**) and heatmap (**b**) to show the differential expressed genes of PANC-1 cells infected by si-Control or si-ITGA2. The blue points represented the downregulated genes, while red points represented the upregulated genes. **c**. Heatmap to show a subset of ITGA2 knockdown regulated genes participated in PD-L1 expression and PD-1 checkpoint pathway of PANC-1 cells. **d**-**f**. Forty-eight hours postinfection, PANC-1, HepG2, SGC-7901, and MDA-MB-231 cells infected with sh-Control or sh-ITGA2s were harvested for RT-PCR analysis (**d**), Western blotting analysis (**e**), and FACS analysis (**f**). Data are shown as means ± SD (*n* = 3). Each sh-ITGA2 group was compared with sh-Control group. Statistical analyses were performed with one-way ANOVA followed by Tukey’s multiple comparison’s tests. *, *P* < 0.05; **, *P* < 0.01; ***, *P* < 0.001. **g**-**h**. Forty-eight hours postinfection, PANC-1, HepG2, SGC-7901, and MDA-MB-231 cells infected with pcDNA3.1 or ITGA2 plasmids were harvested for RT-PCR analysis (**g**) and Western blotting analysis (**h**). Data are shown as means ± SD (*n* = 3–5). ITGA2 overexppression groups were compared with pcDNA 3.1 transfection group. Statistical analyses were performed with one-way ANOVA followed by Tukey’s multiple comparison’s tests. Compared groups were shown in the figures. **, *P* < 0.01; ***, *P* < 0.001

Because ITGA2 is critical for promoting cancer cell progression in vivo and in vitro, we employed RNA-seq analysis to identify the underlying mechanism of how ITGA2 regulates carcinogenesis. Our results identified 427 up-regulated and 365 down-regulated genes after knocking down ITGA2 in PANC-1 cells (Fig. 5)a,b. The pathway analysis of the 40 most significantly regulated genes indicated that ITGA2 repression occurred in the PD-1 checkpoint pathway in cancer cells. Interestingly, the expression of PD-L1 was down-regulated by ITGA2 inhibition (Fig. 5)c. Consistent with the finding from RNA-seq analysis, ITGA2 silencing by two independent sh-RNAs decreased PD-L1 expression in pancreatic, breast, gastric, and liver cancer cells (Fig. 5)d,f. Also, the expression of PD-L1 was up-regulated at both the mRNA and protein levels in cancer cells with overexpressed ITGA2 (Fig. 5g,h). Per all these results, ITGA2 transcriptionally regulates PD-L1 expression in various types of cancer cells.

### The expression of ITGA2 correlates positively with PD-L1 levels in cancer patient specimens

**Fig. 6:**
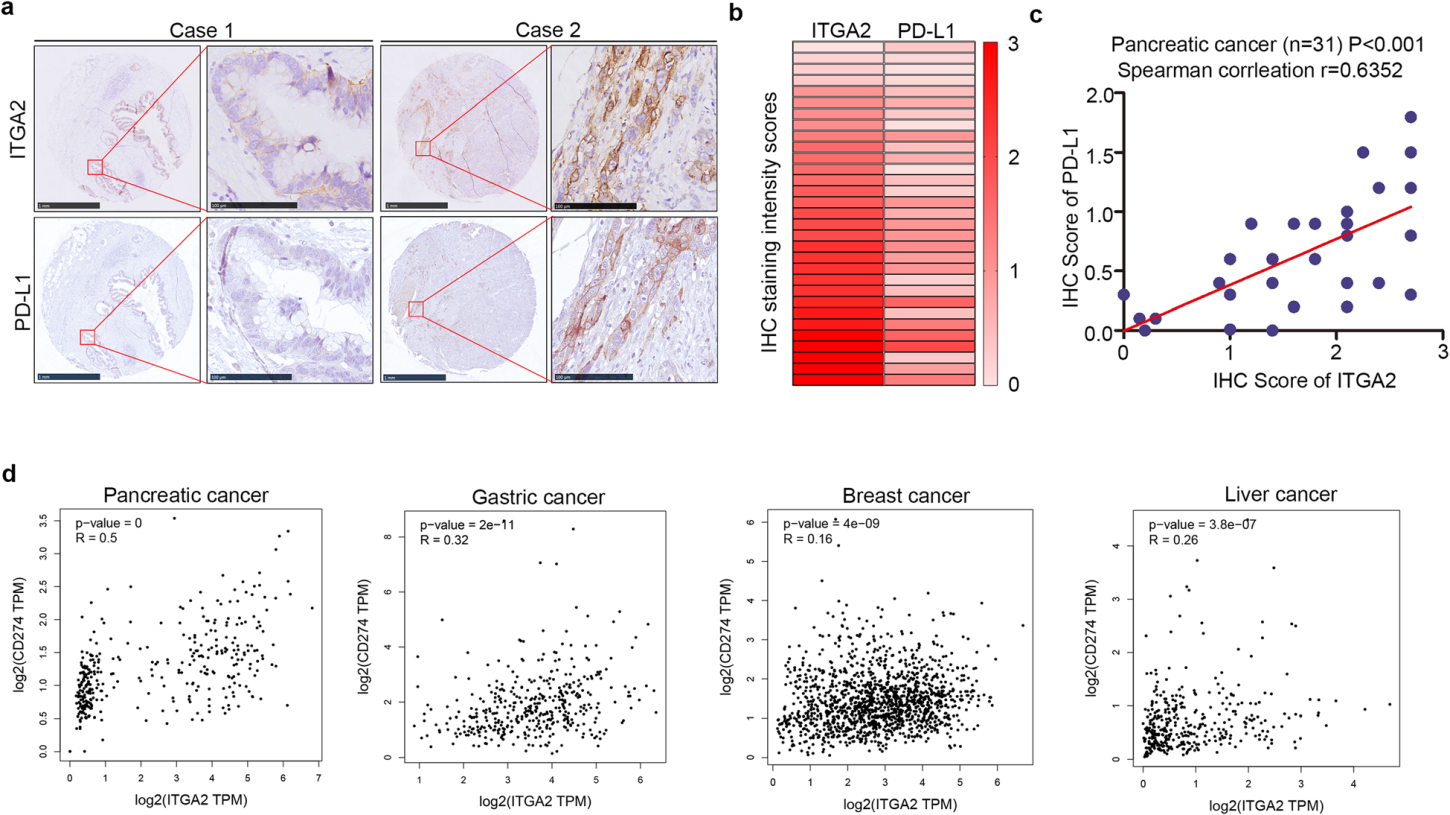
The expression of ITGA2 was positively correlated with PD-L1 in malignant cancer patient specimens. **a**. IHC Images of ITGA2 and PD-L1 staining using TMA tissue sections (*n* = 31 PDAC). The scale bars were shown as indicated. **b** and **c**. Heatmap (**b**) and dot plot (**c**) to show the correlation of IHC scores for the expression of the PD-L1 and ITGA2 proteins in PDAC patient specimens. (r = 0.6352 for spearman correlation coefficients, *P* < 0.001). **d**. The GEPIA web tool was searched for the correlation between the expression of PD-L1 and ITGA2 in mRNA levels in malignant cancer samples. *P* values as indicated in the figure

To determine the clinical relationship between ITGA2 and PD-L1 in human cancer specimens, IHC analysis of the protein expression of ITGA2 and PD-L1 was conducted in a cohort of PDAC patients (*n* = 31). The result showed that there was a positive correlation between the protein levels of ITGA2 and PD-L1 expression in PDAC specimens (Spearman correlation r = 0.6352, *P* < 0.001) (Fig. 6a,c). Furthermore, using the GEPIA web tool, we found that the mRNA expression levels of ITGA2 correlated positively with PD-L1 levels in several malignant tumors, including pancreatic, liver, gastric, and breast cancers (Fig. 6d). Therefore, our data suggest that the level of ITGA2 in cancer specimens has a positive correlation with PD-L1 expression.

### Knocking down Itga2 improves the efficacy of immune checkpoint blockade therapy

**Fig. 7:**
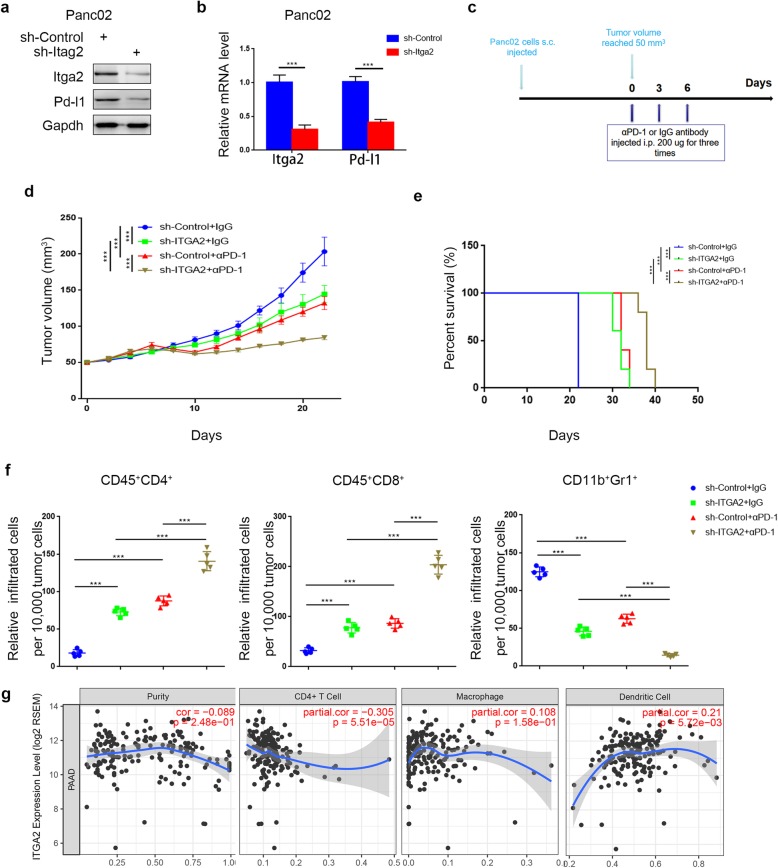
Knockdown of Itga2 improved the efficacy of immune checkpoint blockade therapy. **a** and **b**. Forty-eight hours postinfection, Panc02 cells infected with sh-Control or sh-Itga2 were harvested for Western blotting analysis (**a**) and RT-PCR analysis (**b**). Data are shown as means ± SD (n = 3). sh-Control group was compared with sh-Itga2 group. Statistical analyses were performed with one-way ANOVA followed by Tukey’s multiple comparison’s tests. ***, *P* < 0.001. **c**. After 72 h of selection with puromycin, 5 × 10^6^ Panc02 cells infected with sh-Control or sh-Itga2 were subcutaneously injected into the right dorsal flank of C57BL/6 mice. Mice with subcutaneous Panc02 tumors (n = 5/group) were treated with anti-PD-1 (200 μg) or nonspecific IgG for three times shown as the schematic diagram (**c**). **d**. The Panc02 tumors growth curves (n = 5/group). Groups were compared with each other. Compared groups were shown in the figures. Statistical analyses were performed with two-way ANOVA followed by Sidak’s multiple comparison’s tests. ***, *P* < 0.001. **e**. Kaplan-Meier percent survival curves for each group with different treatments. Groups were compared with each other. Compared groups were shown in the figures. Statistical analyses were performed with Gehan-Breslow-Wilcoxon test. ***, *P* < 0.001. **f**. At the end of treatment, the tumors excised from the mice were dissociated, and tumor cells were harvested for Flow cytometry analysis to detect the numbers of TILs. All data are shown as means ± SD (n = 5). Groups were compared with each other. Compared groups were shown in the figures. Statistical analyses were performed with one-way ANOVA followed by Tukey’s multiple comparison’s tests. *** *P* < 0.001. **g**. The immune infiltration level based on the expression of ITGA2 was searched by the TIMER web tool. *P* values as indicated

As demonstrated above, ITGA2 regulates the expression of PD-L1 in solid cancer cells. So, we assessed the same phenomenon in vivo. Panc02 cells, the murine pancreatic cancer cell lines, were infected with sh-Control or sh-Itga2. In agreement with previous findings, Western blot and RT-PCR analyses showed that Itga2 silencing resulted in decreasing expression of Pd-l1 in Panc02 cells (Fig. 7a,b). After subcutaneous injections of Panc02 cells into the left flank of C57BL immune-proficient mice, the mice were treated with or without anti-PD-1 antibody according to the procedure shown in Fig. 7c. Consistent with the inhibition of cell proliferation by knocking down ITGA2 in human PDAC cells (Fig. 2 and Fig. 4), Itga2 repression inhibited tumor growth in murine Panc02 cells in vivo (Fig. 7d,e). Due to the reduction of Pd-l1 expression after Itga2 suppression, anti-PD-1 antibody treatment manifested a stronger anti-tumor effect in the Itga2 knockdown group by slowing down tumor growth and improving the survival rate of tumor-bearing mice (Fig. 7d,e). The body weights, though, were not significantly different between the different treatments (Additional file 1: Fig. S4b). Flow cytometry analysis of tumors excised from the mice at the end of treatment revealed that the populations of tumor-infiltrated CD45^+^CD8^+^ T-cells and CD45^+^CD4^+^ T-cells increased after Itga2 knockdown, while the population of CD11b^+^Gr1^+^ myeloid cells decreased (Fig. 7f). Meanwhile, the combined therapy of anti-PD-1 antibody and Itga2-knockdown increased the populations of tumor-infiltrated CD45^+^CD8^+^ T-cells and CD45^+^CD4^+^ T-cells further but reduced that of myeloid-infiltrated CD11b^+^Gr1^+^ cells in tumors (Fig. 7f). Using the TIMER web tool [25] to study the immune infiltration level also showed that the expression of ITGA2 correlated positively with dendritic cell infiltration levels and negatively with CD4+ T cell infiltration levels (Fig. 7g). These results suggest that blocking ITGA2 could inhibit cancer-associated immunosuppression and improve the efficacy of immune checkpoint blockade therapy through the downregulation of PD-L1 in vivo.

### ITGA2 increases PD-L1 expression by up-regulating the phosphorylation of STAT3 in cancer cells

**Fig. 8:**
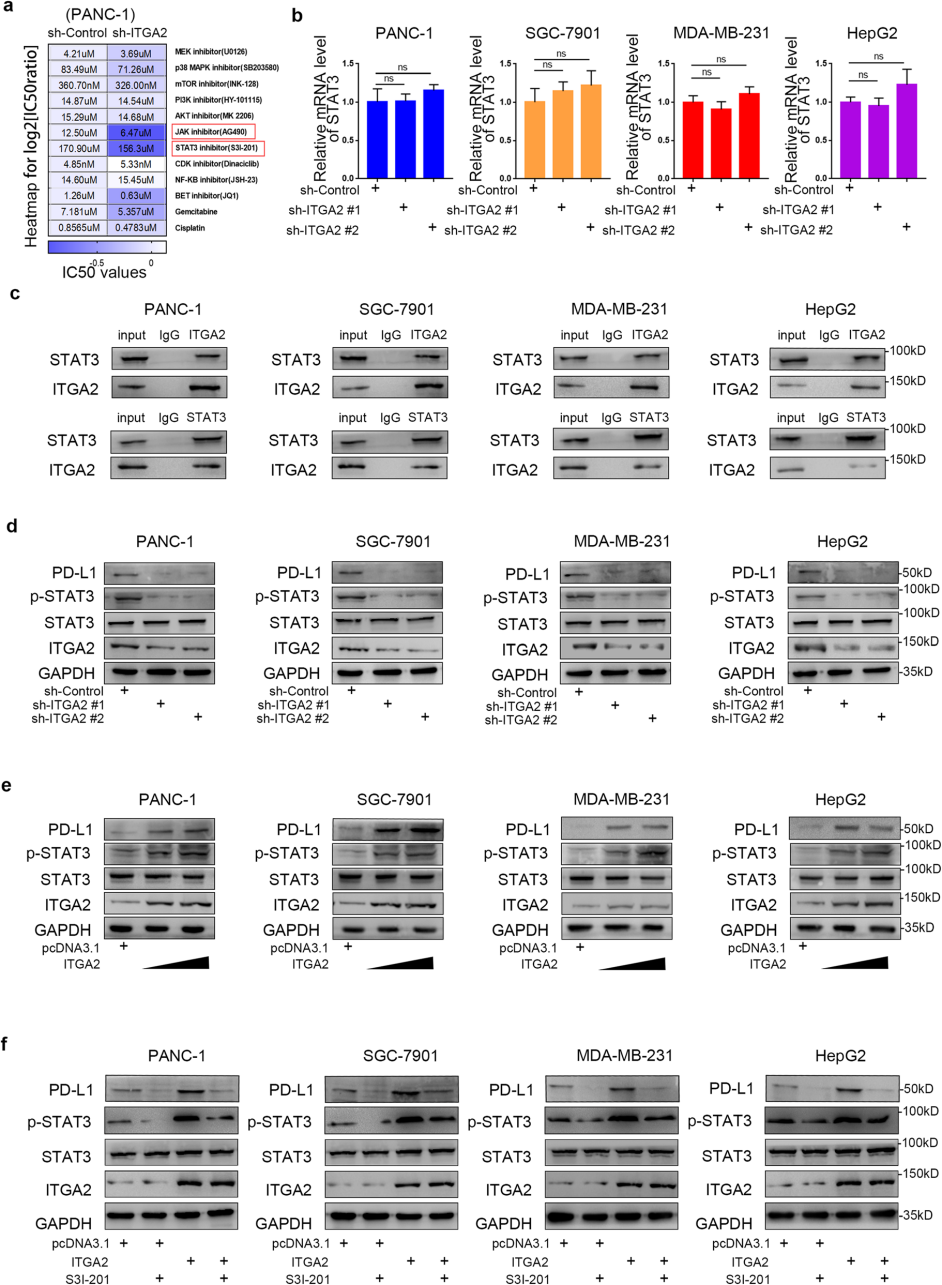
ITGA2 increases PD-L1 expression via up-regulating the phosphorylation of STAT3 in cancer cells. **a**. MTS assay was carried out to measure the cell viability of PANC-1 cells infected with sh-Control or sh-ITGA2 #1 and treated with different chemicals. IC50 was shown as indicated. Heatmap to show the normalized IC50 ratio (log2[IC50ratio]). **b**. Forty-eight hours postinfection, PANC-1, HepG2, SGC-7901, and MDA-MB-231 cells infected with sh-Control or sh-ITGA2 were harvested for RT-PCR analysis. All data were shown as mean ± SD (*n* = 3). Each sh-ITGA2 group was compared with sh-Control group. Statistical analyses were performed with one-way ANOVA followed by Tukey’s multiple comparison’s tests. ns, not significant. **c**. Western blot analysis for IP samples of PANC-1, HepG2, SGC-7901, and MDA-MB-231 cells. **d**. Forty-eight hours postinfection, PANC-1, HepG2, SGC-7901, and MDA-MB-231 cells infected with sh-Control or sh-ITGA2 were harvested for Western blotting analysis. **e** and **f**. Forty-eight hours postinfection, PANC-1, HepG2, SGC-7901, and MDA-MB-231 cells infected with or without ITGA2 plasmids (**e**) and treated with or without STAT3 inhibitor (**f**) were harvested for Western blotting analysis.

Given that ITGA2 promoted PD-L1 expression in cancer cells, we wanted to identify the underlying mechanism of the process. Interestingly,we detected drug sensitivity (IC50 values) from PANC-1 cells to some cancer-related pathway small inhibitors in the sh-Control and sh-ITGA2 groups (Fig. 8a). The results showed that the JAK and STAT3 inhibitors increased viability loss in ITGA2-knockdown cells further (Fig. 8a), indicating that ITGA2 might be involved in modulating the STAT3-related signaling pathway in cells.

To determine the relationship between ITGA2 and the STAT3 signaling pathway, we, first of all, examined the mRNA expression level of STAT3 in cancer cells infected with sh-Control or sh-ITGA2. The result revealed that there was no noticeable change in the mRNA expression of STAT3 (Fig. 8b), which pointed to ITGA2 not regulating the expression of STAT3 at the transcriptional level. Per the immunoprecipitation assay (IP), ITGA2 interacted with STAT3 in various types of cancer cells. (Fig. 8c). Furthermore, Western blot analysis established that there was no regulation of STAT3 expression at the protein level (Fig. 8d). Consequently, we could not link the role of ITGA2 in regulating PD-L1 expression to the regulation of STAT3 expression.

L.L. Bu et al. reported that the expression of PD-L1 was regulated by p-STAT3 in a dose-dependent manner [26]. Herein, in our next step, we hypothesized that p-STAT3 was the key protein that mediated PD-L1 expression induced by ITGA2. To test this hypothesis, we examined the changes in phospho-STAT3 in cancer cells. We found that knocking down ITGA2 suppressed the phosphorylation level of STAT3 (Fig. 8d). Similarly, the overexpression of ITGA2 increased the phosphorylation level of STAT3 and protein level of PD-L1 in cancer cells (Fig. 8e), and these effects were diminished by STAT3 inhibitor treatment (Fig. 8f). More importantly, the activation of the STAT3 pathway while knocking down ITGA2 also upregulated the expression level of PD-L1 (Additional file 1: Fig. S5). Overall, our findings suggest that ITGA2 enhances STAT3 phosphorylation by serving as the origin of the transcription and translation of PD-L1 in cancer cells.

## Discussion

Integrins are essential in mediating cell-matrix and cell-cell interactions as heterodimeric proteins on the cell surface that have been confirmed to participate in the processes of tumor initiation, progression, and metastasis [27, 28]. As a subunit of integrin, ITGA2 is closely associated with tumor cell proliferation, migration, and invasion [29]. The high expression of ITGA2 contributes to the reduced survival rate of solid cancer. Consistent with this fact, our results revealed that ITGA2 was abnormally over-expressed in many malignant tumors, including pancreatic cancer, gastric cancer, liver cancer, and breast cancer. Furthermore, our data demonstrated that knocking down ITGA2 inhibited cancer cell proliferation (Fig. 2 and Fig. 4), but the overexpression of ITGA2 promoted tumor cell proliferation (Fig. 3 and Fig. 4). Interestingly, our data also established that the expression of ITGA2 was most up-regulated in PDAC, suggesting that ITGA2 might play an essential role in the pathogenesis of PDAC. Additionally, our findings point to ITGA2 acting as an oncogenic protein to promote the progression of solid cancer, especially pancreatic cancer.

Cancer is a complex disease process that involves the interaction between the tumor and the host immune system [30]. Several studies have reported that the number of immune infiltration cells increases in cancer, but most of the augmented immune cells are immunosuppression-related cells, such as regulatory T-cell (Treg), M2 macrophages (M2), and myeloid-derived suppressor cells (MDSC) [31–33]. Additionally, increased immunosuppression-related cells correlate with poor survival in various types of malignant tumors, but Treg and MDSC depletion therapy can delay tumor growth in vivo [33, 34]. Per these reports, the dynamic network among immune cells themselves suggests a key role of the network in tumor progression and behaviors. Understanding the mechanism of how to down-regulate the infiltration of tumor suppression immune cells and up-regulate tumor-killing immune cells is essential to improving the efficiency of immune therapy. In this study, we found that ITGA2 inhibition increased the ratio of tumor-killing lymphocytes and decreased the proportion of immunosuppression-related cells in tumors (Fig. 7), which suggests that ITGA2 might be an ideal candidate for immune therapy.

The PD-1/PD-L1 immune checkpoint blockade allows to potentiate antitumor immunity by inhibiting immunosuppressive signals from co-inhibitory molecules, and this has achieved great success in many malignant tumors [35–39]. The interaction between PD-L1 on tumor cells and PD-1 on T-cells inhibits the biological functioning of antigen-specific CD8^+^T cells and conduces cancer cells to escape immune destruction [40, 41]. Therefore, how to inhibit the PD-L1 expression of cancer cells is essential to cancer immune therapy. Encouragingly, Guo P et al. reported that targeting ITGA2 improved the antitumor efficacy of immune therapy in glioblastoma multiforme (GBM) [42]. Here, our RNA-seq analysis showed that PD-L1 expression and the PD-1 checkpoint pathway were selectively downregulated after knocking down ITGA2 in PANC-1 cells. Meanwhile, we also found that ITGA2 transcriptionally increased PD-L1 expression in multiple types of cancer cells. Moreover, ITGA2 repression enhanced the tumor inhibition effect of the anti-PD-1 antibody in vivo. These results suggest that ITGA2 might be critical in modulating the anti-tumor efficiency of immune checkpoint-based therapy.

It has been reported that miR-16-5p inhibits tumor progression by down-regulating ITGA2 in colorectal cancer [43]. ADAR1 also reportedly enhances hepatocellular carcinoma (HCC) metastasis by promoting tumor cell adhesion to ECM via increasing ITGA2 expression [8, 42]. Furthermore, blocking ITGA2 inhibits gastric cancer and glioblastoma (GBM) cell migration of [42]. However, the specific biological mechanism of how the expression of ITGA2 correlates with the progression of cancer and the blockade of ITGA2 inhibits the migration of cancer cells has remained unknown for a long time. Here, we demonstrated that ITGA2 might regulate cancer cell proliferation and migration by regulating the expression of PD-L1.

Recently, several studies have shown that various transcriptional factors, such as STAT1 [44], STAT3 [45], BRD4 [23], NF-kB [18], PI3K/AKT/mTOR [46], and MEK1/2/ERK1/2 [47], promoted PD-L1 transcription in cancer cells. By carrying out a drug sensitivity assay and several verification experiments, we determined that ITGA2 bound with STAT3 to initiate the transcription of PD-L1 by increasing the phosphorylation level of STAT3 in cancer cells. Therefore, ITGA2 is potentially a new regulator of PD-L1 expression, and this regulation might be attributed to the activation of the STAT3 signaling pathway. All our findings suggest that more research has to be carried out on the use of ITGA2 as a novel immune therapeutic target for malignant tumors.

## Conclusion

We investigated the specific biological role of ITGA2 in cancer in this research, and our results revealed that ITGA2 was abnormally overexpressed and significantly associated with unfavorable survival rates in several malignant tumors. Moreover, we demonstrated that ITGA2 played an essential role in promoting cell proliferation and invasion in cancer. Our findings also showed that blocking ITGA2 improved tumor immune responses by decreasing the phosphorylation level of STAT3 and suppressing PD-L1 expression in vivo. Collectively, ITGA2 could serve as a novel prognostic biomarker of solid cancer and a novel target for immune checkpoint blockade therapy.

## Supplementary information

**Additional file 1: Figure S1.** Overexpressed ITGA2 correlated with malignant cancer progression and poor prognosis. **a.** The overall survival of the patients with LIHC were computed with the GEPIA web tool (*P* = 0.022, HR = 1.5).**b.** The disease-free survival of the patients with LIHC were computed with the GEPIA web tool (*P* = 0.03, HR = 1.4).**c.** The overall survival of the patients with LIHC was computed with the Human Protein Atlas (*P* < 0.001). **Figure S2.** Silencing ITGA2 suppresses the invaded ability of malignant cancer in vitro. PANC-1, HepG2, SGC-7901, and MDA-MB-231 cells were infected with sh-Control or sh-ITGA2 #1. The cells were harvested for invasion assay after forty-eight hours culturing. Representative images of invaded cells were shown based on a transwell assay. Each bar represents the mean ± SD of three independent experiments. Each sh-ITGA2 group was compared with sh-Control group. Statistical analyses were performed with one-way ANOVA followed by Tukey’s multiple comparison’s tests.. **, *P* < 0.01; ***, *P* < 0.001. **Figure S3**. Overpressed ITGA2 promotes the invaded ability of malignant cancer in vitro.PANC-1, HepG2, SGC-7901, and MDA-MB-231 cells were infected with pcDNA3.1 or ITGA2 plasmid. The cells were harvested for invasion assay after forty-eight hours culturing. Representative images of invaded cells were shown based on a transwell assay. Each bar represents the mean ± SD of three independent experiments. ITGA2 overexppression groups were compared with pcDNA3.1 transfection group. Statistical analyses were performed with one-way ANOVA followed by Tukey’s multiple comparison’s tests. Compared groups were shown in the figures. **, P < 0.01; ***, P < 0.001. **Figure S4**. The body mass changes for PDAC xenografts nude mice and Syngeneic tumor model C57BL/6 mice a.The body mass changes for PDAC xenografts nude mice. The result showed that different treatments didn’t significantly affect the mouse mass and they were not toxic. ns, not significant. b.The body mass changes for Syngeneic tumor model C57BL/6 mice. The result showed that different treatments didn’t significantly affect the mouse mass and they were not toxic. ns, not significant. **Figure S5**. STAT3 agonists (IL-6) can reverse the downregulation of PD-L1 mediated by ITGA2 knockdown in cancer cells.Forty-eight hours postinfection, PANC-1, HepG2, SGC-7901, and MDA-MB-231 cells infected with or without sh-ITGA2 and IL-6 were harvested for Western blotting analysis. **Table S1** Sequences of RT-qPCR primers. **Table S2**: Sequences of gene-specific shRNAs and siRNAs.

## Data Availability

Please contact the corresponding author (Xin Jin, jinxinunion@hust.edu.cn) for data requests.

## Abbreviations

BRCA: Breast invasive carcinoma
DFS: Disease-free survival
GEPIA: Gene Expression Profiling Interaction Analysis
IHC: Immunohistochemistry
ITGA2: Integrin alpha 2
LIHC: Liver hepatocellular carcinoma
M2: M2 macrophages
MDSC: Myeloid-derived suppressor cells.
OS: Overall survival
PAAD: Pancreatic adenocarcinoma
PDAC: Pancreatic ductal adenocarcinoma
PD-L1: Programmed death-ligand 1
STAD: Stomach adenocarcinoma
STAT3: Signal transducer and activator of transcription 3;
TMA: Tissue microarray
Treg: Regulatory T-cell
