# Supplementary material for: Overexpressed ITGA2 promotes malignant tumor aggression by up-regulating PD-L1 expression through the activation of the STAT3 signaling pathway
Source: J Exp Clin Cancer Res. 2019 Dec 9;38:485. doi: 10.1186/s13046-019-1496-1 (PMC6902401; doi:10.1186/s13046-019-1496-1)
Supplement: Supplementary file 1 — Additional file 1: Figure S1. Overexpressed ITGA2 correlated with malignant cancer progression and poor prognosis. a. The overall survival of the patients with LIHC were computed with the GEPIA web tool (P = 0.022, HR = 1.5).b. The disease-free survival of the patients with LIHC were computed with the GEPIA web tool (P = 0.03, HR = 1.4).c. The overall survival of the patients with LIHC was computed with the Human Protein Atlas (P < 0.001). Figure S2. Silencing ITGA2 suppresses the invaded ability of malignant cancer in vitro. PANC-1, HepG2, SGC-7901, and MDA-MB-231 cells were infected with sh-Control or sh-ITGA2 #1. The cells were harvested for invasion assay after forty-eight hours culturing. Representative images of invaded cells were shown based on a transwell assay. Each bar represents the mean ± SD of three independent experiments. Each sh-ITGA2 group was compared with sh-Control group. Statistical analyses were performed with one-way ANOVA followed by Tukey’s multiple comparison’s tests.. **, P < 0.01; ***, P < 0.001. Figure S3. Overpressed ITGA2 promotes the invaded ability of malignant cancer in vitro.PANC-1, HepG2, SGC-7901, and MDA-MB-231 cells were infected with pcDNA3.1 or ITGA2 plasmid. The cells were harvested for invasion assay after forty-eight hours culturing. Representative images of invaded cells were shown based on a transwell assay. Each bar represents the mean ± SD of three independent experiments. ITGA2 overexppression groups were compared with pcDNA3.1 transfection group. Statistical analyses were performed with one-way ANOVA followed by Tukey’s multiple comparison’s tests. Compared groups were shown in the figures. **, P < 0.01; ***, P < 0.001. Figure S4. The body mass changes for PDAC xenografts nude mice and Syngeneic tumor model C57BL/6 mice a.The body mass changes for PDAC xenografts nude mice. The result showed that different treatments didn’t significantly affect the mouse mass and they were not toxic. ns, not significant. b.The body m [file 13046_2019_1496_MOESM1_ESM.docx]

**The overexpressed ITGA2 promotes malignant tumor aggressiveness by up-regulating PD-L1 expression through activation STAT3 signal pathway**

Dianyun Ren, Jingyuan Zhao , Yan Sun , Dan Li , Zibo Meng , Bo Wang , Ping Fan，Zhiqiang Liu, Xin Jin, Heshui Wu

**
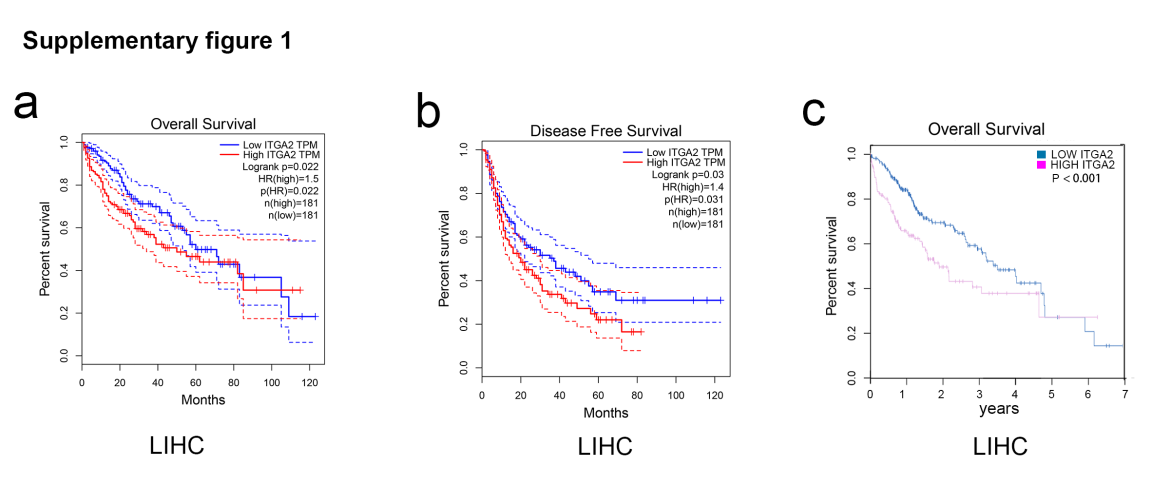
**

**Supplementary figure 1. Overexpressed ITGA2 correlated with malignant cancer progression and poor prognosis.**

**a.** The overall survival of the patients with LIHC were computed with the GEPIA web tool (P = 0.022, HR = 1.5).

**b.** The disease-free survival of the patients with LIHC were computed with the GEPIA web tool (P = 0.03, HR = 1.4).

**c.** The overall survival of the patients with LIHC was computed with the Human Protein Atlas (P < 0.001).


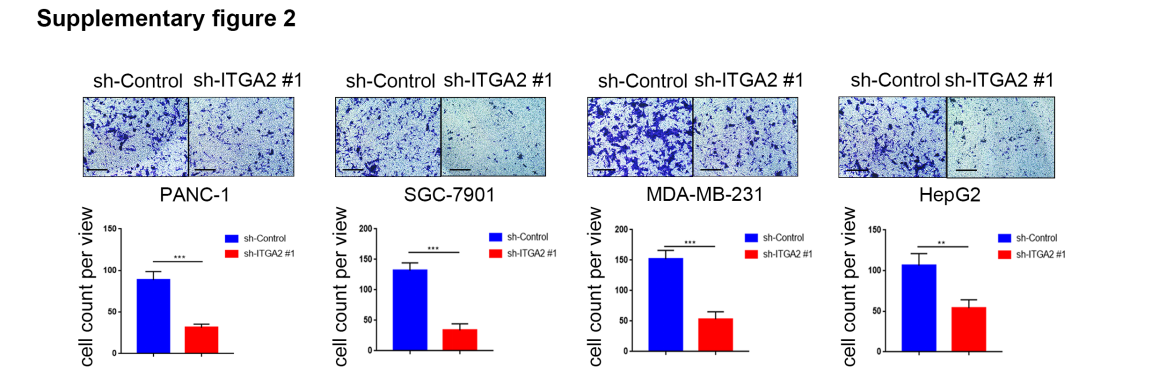


**Supplementary figure 2. Silencing ITGA2 suppresses the invaded ability of malignant cancer in vitro**

PANC-1, HepG2, SGC-7901, and MDA-MB-231 cells were infected with sh-Control or sh-ITGA2 #1. The cells were harvested for invasion assay after forty-eight hours culturing. Representative images of invaded cells were shown based on a transwell assay. Each bar represents the mean ± SD of three independent experiments. Each sh-ITGA2 group was compared with sh-Control group. Statistical analyses were performed with one-way ANOVA followed by Tukey's multiple comparison's tests.. **, P < 0.01; ***, P < 0.001.

**
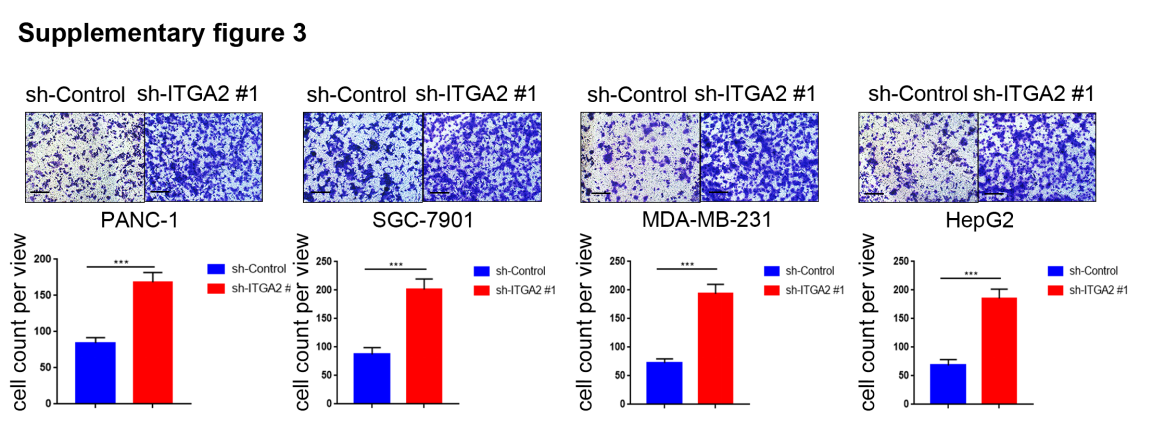
**

**Supplementary figure 3. Overpressed ITGA2 promotes the invaded ability of malignant cancer in vitro.**

PANC-1, HepG2, SGC-7901, and MDA-MB-231 cells were infected with pcDNA3.1 or ITGA2 plasmid. The cells were harvested for invasion assay after forty-eight hours culturing. Representative images of invaded cells were shown based on a transwell assay. Each bar represents the mean ± SD of three independent experiments. ITGA2 overexppression groups were compared with pcDNA3.1 transfection group. Statistical analyses were performed with one-way ANOVA followed by Tukey's multiple comparison's tests.Compared groups were shown in the figures. **, P < 0.01; ***, P < 0.001.

**
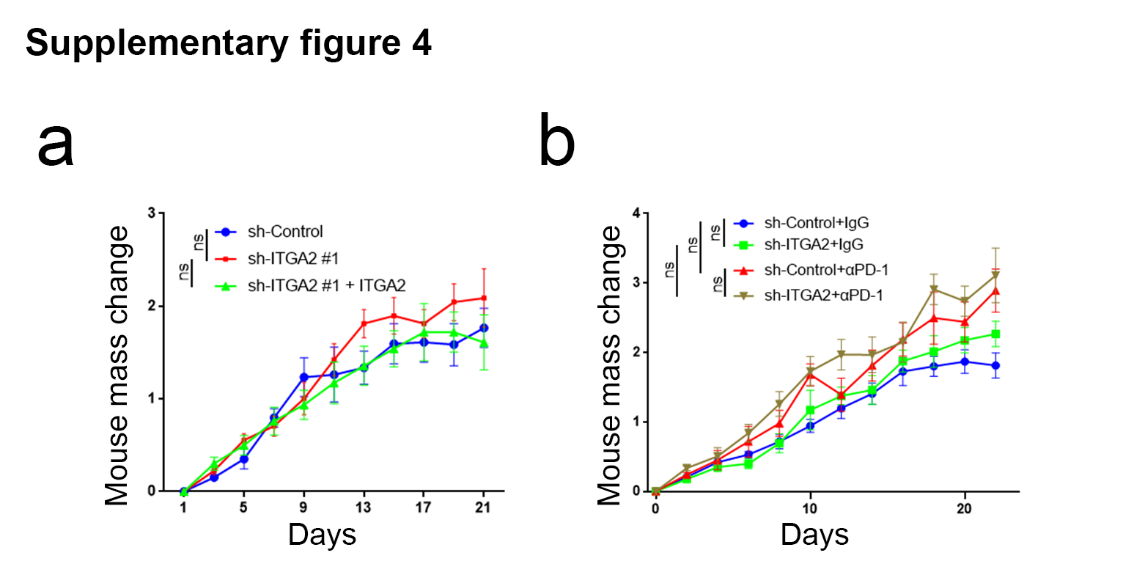
**

**Supplementary figure 4. The body mass changes for PDAC xenografts nude mice and Syngeneic tumor model C57BL/6 mice.**

1. The body mass changes for PDAC xenografts nude mice. The result showed that different treatments didn’t significantly affect the mouse mass and they were not toxic. ns, not significant.
2. The body mass changes for Syngeneic tumor model C57BL/6 mice. The result showed that different treatments didn’t significantly affect the mouse mass and they were not toxic. ns, not significant.

**
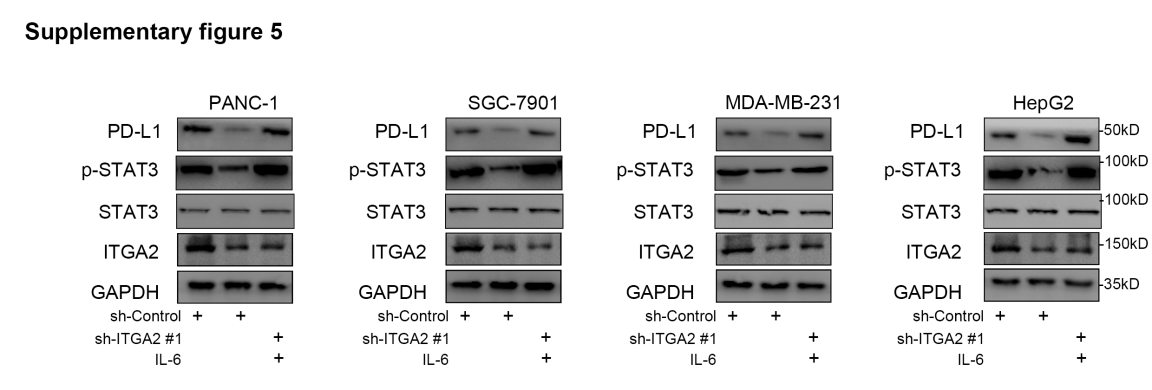
Supplementary figure 5. STAT3 agonists (IL-6) can reverse the downregulation of PD-L1 mediated by ITGA2 knockdown in cancer cells.**

Forty-eight hours postinfection, PANC-1, HepG2, SGC-7901, and MDA-MB-231 cells infected with or without sh-ITGA2 and IL-6 were harvested for Western blotting analysis.

**Supplementary** **Table 1: Sequences of RT-qPCR primers**

| **Species** | **Gene** | **Forward (5’-3’)** | **Reverse (5’-3’)** |
| --- | --- | --- | --- |
| Human | GAPDH | ACCCAGAAGACTGTGGATGG | TTCAGCTCAGGGATGACCTT |
| Human | ITGA2 | GGGAATCAGTATTACACAACGGG | CCACAACATCTATGAGGGAAGGG |
| Human | PD-L1 | GGTGCCGACTACAAGCGAAT | AGCCCTCAGCCTGACATGTC |
| Human | STAT3 | CAGCAGCTTGACACACGGTA | AAACACCAAAGTGGCATGTGA |
| Mouse | Gapdh | AGGTTGTCTCCTGCGACTTCA | GGGTGGTCCAGGGTTTCTTACT |
| Mouse | Pd-l1 | AATGCTGCCCTTCAGATCAC | ATAACCCTCGGCCTGACATA |
| Mouse | Itga2 | TGTCTGGCGTATAATGTTGGC | CTTGTGGGTTCGTAAGCTGCT |

**Supplementary Table 2: Sequences of gene-specific shRNAs and siRNAs**

| si-Control | purchased from RIBOBIO |
| --- | --- |
| si-ITGA2 #1 | CAGCCGCTACTTCCAATAT |
| si-ITGA2 #2 | CAGGACTGGACATATCTGC |
| sh-ITGA2 #1 | CCGGCCGGCCAGATAGTGCTATATACTCGAGTATATAGCACTATCTGGCCGGTTTTTG |
| sh-ITGA2 #2 | CCGGATGGCAATATCACGGTTATTCCTCGAGGAATAACCGTGATATTGCCATTTTTTG |
| sh-Itga2 #1 | CCGGCGGCTGCTAATGCTAGTTCAACTCGAGTTGAACTAGCATTAGCAGCCGTTTTTG |
| sh-Itga2 #2 | CCGGTCGCAAGAGACTACGCTTATTCTCGAGAATAAGCGTAGTCTCTTGCGATTTTTG |
